# Intermediate gastropod hosts of major feline cardiopulmonary nematodes in an area of wildcat and domestic cat sympatry in Greece

**DOI:** 10.1186/s13071-020-04213-z

**Published:** 2020-07-10

**Authors:** Dimitris Dimzas, Simone Morelli, Donato Traversa, Angela Di Cesare, Yoo Ree Van Bourgonie, Karin Breugelmans, Thierry Backeljau, Antonio Frangipane di Regalbono, Anastasia Diakou

**Affiliations:** 1 School of Veterinary Medicine, Faculty of Health Sciences, Aristotle University of Thessaloniki, 54124 Thessaloniki, Greece; 2 Faculty of Veterinary Medicine, Teaching Veterinary Hospital, 64100 Teramo, Italy; 3 Royal Belgian Institute of Natural Sciences (BopCo - LifeWatch Belgium), 1000 Brussels, Belgium; 4 Evolutionary Ecology Group, Department of Biology, University of Antwerp, 2610 Antwerp, Belgium; 5 Department of Animal Medicine, Production and Health, University of Padua, 35020 Padua, Italy

**Keywords:** *Aelurostrongylus abstrusus*, *Angiostrongylus chabaudi*, Cardiopulmonary parasites, Domestic cat, Gastropods, Slugs, Snails, *Troglostrongylus brevior*, Wildcat

## Abstract

**Background:**

The metastrongyloid nematodes *Aelurostrongylus abstrusus*, *Troglostrongylus brevior* and *Angiostrongylus chabaudi* are cardiopulmonary parasites affecting domestic cats (*Felis catus*) and wildcats (*Felis silvestris*). Although knowledge on these nematodes has been improved in the past years, gaps in our knowledge of their distribution and role of gastropods as intermediate hosts in Europe still exist. This study reports on the presence of these nematodes and their intermediate hosts in an area in Greece where domestic cats and wildcats occur in sympatry.

**Methods:**

Terrestrial gastropods were collected in the field and identified morphologically and by mitochondrial DNA-sequence analysis. Metastrongyloid larvae were detected by artificial digestion, morphologically identified to the species and stage level and their identity was molecularly confirmed.

**Results:**

*Aelurostrongylus abstrusus* was found in the snails *Massylaea vermiculata* and *Helix lucorum*, *T. brevior* in the slug *Tandonia* sp., and *A. chabaudi* in the slug *Limax* sp. and the snails *H. lucorum* and *M. vermiculata.*

**Conclusions:**

To the best of our knowledge this study provides the first reports of (i) terrestrial gastropods being naturally infected with *A. chabaudi*, (ii) *T. brevior* naturally infecting terrestrial gastropods in Europe, and (iii) *A. abstrusus* naturally infecting terrestrial gastropods in Greece. Furthermore, the present study describes for the first time developmental stages of *A. chabaudi* and *T. brevior* in naturally infected gastropods. The biological characteristics of various intermediate gastropod hosts that could influence the distribution and expansion of feline cardiopulmonary nematodes are discussed, along with epizootiological implications and perspectives.
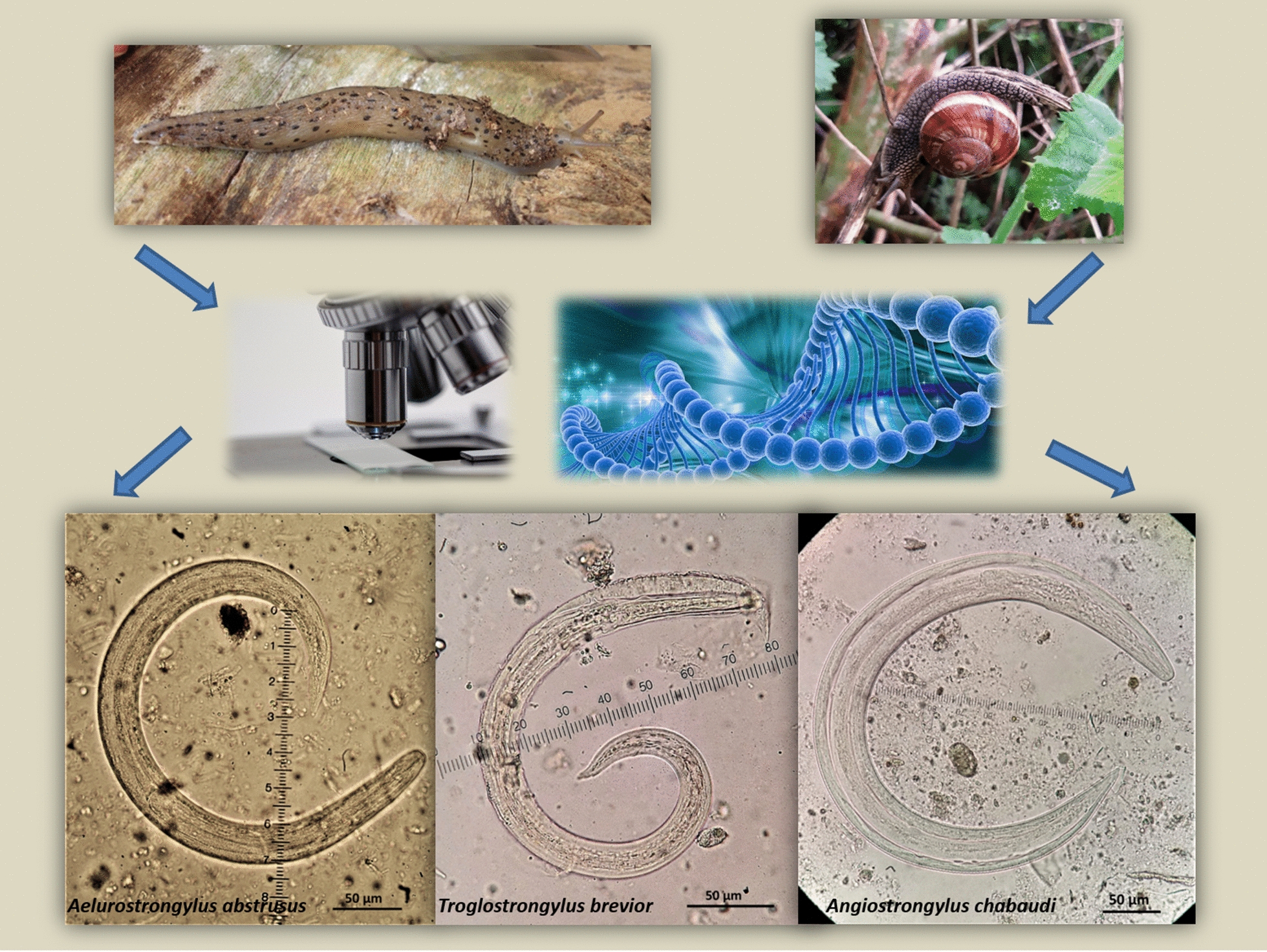

## Background

Three nematodes belonging to the superfamily Metastrongyloidea (order Strongylida), i.e. *Aelurostrongylus abstrusus*, *Troglostrongylus brevior* and *Angiostrongylus chabaudi*, are cardiopulmonary parasites affecting the domestic cat (*Felis catus*), the European wildcat (*Felis silvestris*) and other wild felids [1, 2]. The life-cycle of these parasites is indirect: felines shed first-stage larvae (L1) that continue their development in a terrestrial gastropod mollusc to develop to second-stage (L2), and subsequently third-stage (L3) larvae, that is infective for the definitive host; paratenic hosts (small vertebrates) also play an important role in the biological cycle of these nematodes [1, 3]. Because of their veterinary significance and apparently expanding distribution both in enzootic and previously non-enzootic regions, these parasites are gaining scientific interest [1, 4–8].

*Aelurostrongylus abstrusus* (Railliet, 1898) (“cat lungworm”) affects domestic cats worldwide and is considered the major felid respiratory parasite [1, 9]. Nevertheless, during the last few years a different lungworm, i.e. *T. brevior*, traditionally considered to be associated only with wild felids, has been increasingly reported in domestic cats [7]. This lungworm was described in 1949 in wild felids from Palestine [10] and later recorded in wildcats in central Italy [11]. In the 21st century, *T. brevior* has been reported in domestic cats from various Mediterranean islands, i.e. Ibiza, Sicily, Sardinia, Crete, Mykonos, Skopelos and Cyprus (see Crisi et al. [7] for details) but also in continental Europe, i.e. Italy [12], Greece [13], Bosnia and Herzegovina [14], Bulgaria, Spain [15] and Romania [16].

In contrast, very little is known about *A. chabaudi* causing feline angiostrongylosis, first identified in European wildcats from central Italy [17]. Since the original description, this parasite has never been reported until recently, when immature adult specimens were found in two domestic cats in Italy [5, 6]. Recently, *A. chabaudi* has been reported in wildcats from Greece, Romania, Bulgaria, Italy and Bosnia and Herzegovina [2, 18–21].

The detection of *A. abstrusus*, *T. brevior* and *A. chabaudi* in both wildcats and domestic cats, along with many other pathological, clinical and molecular data [1, 22, 23], indicate possible routes of cross transmission between these feline species, especially in areas where domestic cats and wildcats co-exist. For example, infections with *T. brevior* in domestic cats are mostly documented in areas within the distributional range of wildcats [6, 24–26]. Analogously, *A. abstrusus* may also infect wildcats in regions with high prevalence in domestic cat populations [12, 21, 23]. Important factors related to the circulation and transmission of these parasites are the distribution and biological features of their intermediate hosts, i.e. terrestrial snails and slugs. These nematodes reach the infective stage in various gastropod species under experimental conditions [27–30]. Furthermore, there are some reports of gastropods naturally infected with *A. abstrusus* and *T. brevior* [31–39]. Conversely, natural infections of gastropods with *A. chabaudi* have not yet been documented, leaving an important gap in the poorly-known life-cycle of this parasite.

In this context, there is a merit in improving knowledge on the role of different mollusc species implicated in the transmission of these parasites to wildcats and domestic cats in natural environments, especially in areas where both felines occur in sympatry. Such data would help to predict the geographical spread of lungworms based on the distribution of their intermediate hosts. Thus, the aim of the present study was to investigate slugs and snails in areas where they could act as the interface for the spillover of metastrongyloid infections between the two felines. Herein we report the first cases of gastropod natural intermediate hosts of feline metastrongyloid nematodes, found in the frame of a larger, ongoing project investigating terrestrial gastropods as intermediate hosts of nematode parasites in Greece.

## Methods

### Study area and sample collection

**Fig. 1:**
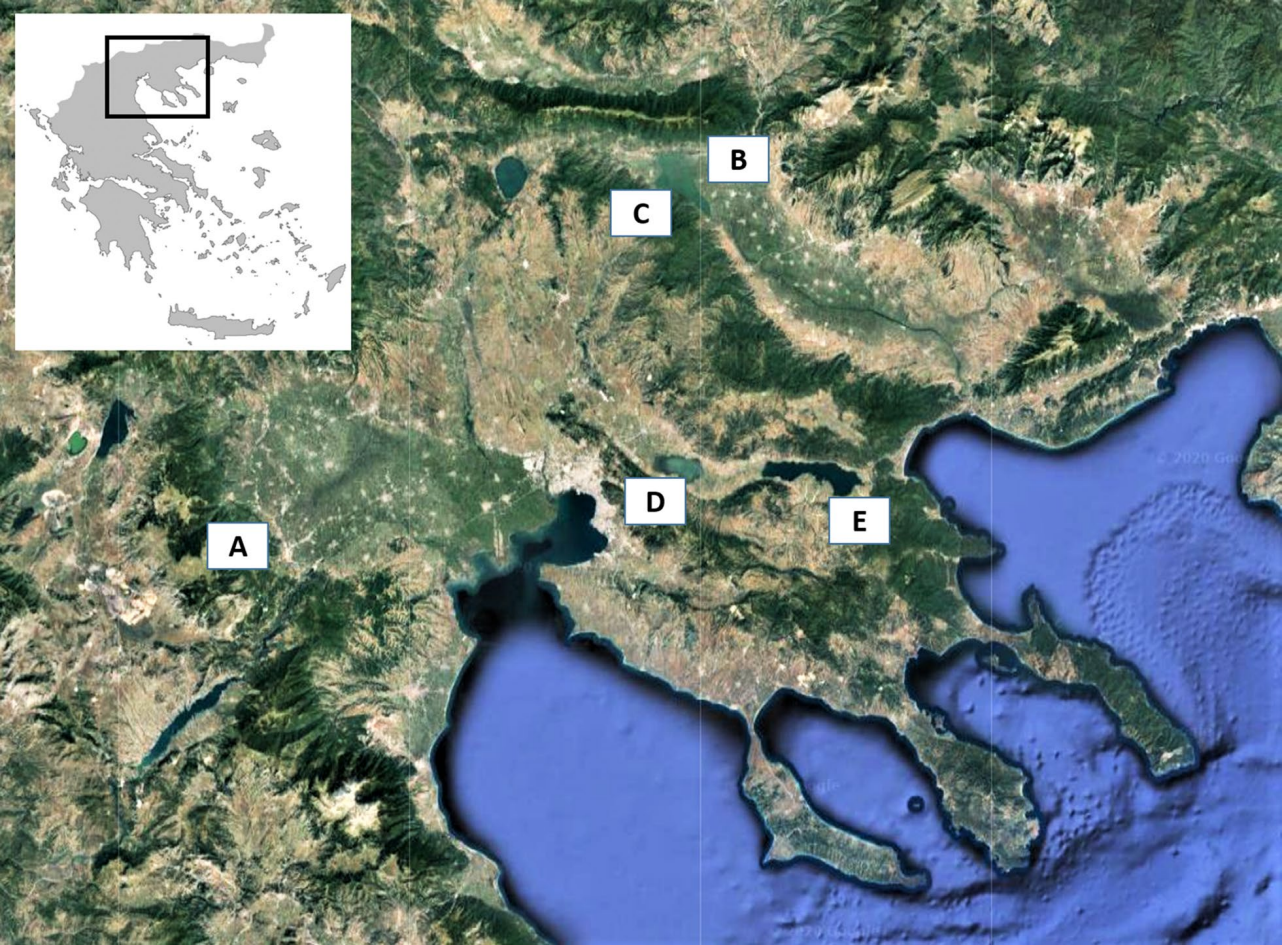
Areas of Greece where terrestrial gastropods infected with feline metastrongyloids where collected. A, Kozani (*Aelurostrongylus abstrusus* in *Helix lucorum*); B, Vyrοnia (*A. abstrusus* in *Massylaea vermiculata*); C, Lake Kerkini (*Troglostrongylus brevior* in *Tandonia* cf. *sowerbyi*); D, Lake Koronia (*Angiostrongylus chabaudi* in *H. lucorum* and *M. vermiculata*); E, Lake Volvi (*A. chabaudi* in *Limax* cf. *conemenosi*)

The gastropods were sampled as a part of an ongoing project investigating terrestrial gastropods as intermediate hosts of parasitic nematodes in Greece. The specimens were collected in five sub-urban, rural and wild habitats (sites A-E, Fig. 1), where wildcats and domestic cats co-exist, in order to investigate slugs and snails in areas where they could act as the interface for the spillover of metastrongyloid infections between the two felines. More precisely, site A (sub-urban area of the city of Kozani), is an area with few houses and plenty of vegetation (grasses, trees, etc.), characterised by a continental climate with dry, cold winters, an average yearly temperature of 13 °C and a relative humidity of 74% [40]. Site B is a typical rural environment at the village Vyronia, and sites C, D and E are close to three important wetlands (Lakes Kerkini, Koronia and Volvi, respectively), that are considered wild habitats, however close to human activities. The climate at Vyronia and around the lakes is a semi-arid Mediterranean climate, with mild to cold winters, an average yearly temperature of 15 °C and annual rainfall between 600–650 mm [40].

Gastropods (both active and hibernating/estivating) were collected by hand from the study areas, placed individually in small plastic bags and tagged with a code that corresponded to a form with the details of the collection (date, coordinates and type of environment). The present data are derived from the examination of 25 snails (8, 7, 3 and 7 from sites A, B, C and D, respectively) and 25 slugs (1, 8, 11 and 5 from cites A, B, C and E, respectively) as preliminary results of the aforementioned broader study. All specimens were cryo-euthanised immediately in a deep freezer and stored frozen at − 20 °C until further examination.

### Identification of gastropods

For each examined gastropod, individual photographs were used for morphological assessment and foot tissue sampling (*c.*2 mm^3^) for DNA-based identification. Furthermore, from each sampling spot whole specimens with the same phenotype were collected and conserved in 70% ethanol for morphological confirmation. The nomenclature of the molluscs applied follows MolluscaBase [41].

DNA-based identification of gastropods was performed on infected specimens and relied on two mtDNA fragments, i.e. the cytochrome *c* oxidase subunit 1 (*cox*1) barcode and *16S* ribosomal DNA (*16S*). Genomic DNA was extracted with the Nucleospin® Tissue Kit (Macherey-Nagel, Düren, Germany) according to the manufacturer’s instructions. The *cox*1 fragment was PCR amplified using the primers LCO1490 (forward) and HCO2198 (reverse) [42] and *16S* using the primers 16Sar (forward) and 16Sbr (reverse) [43]. PCR was carried out in 12.5 µl reaction volumes containing 0.25 µl of dNTP mix (containing 10 mM of each dNTP), 1.25 µl of 2 µM of each primer, 0.05 µl of 10 U/µl Platinum® *Taq* DNA Polymerase (Thermo Fisher Scientific, Waltham, USA), 1.25 µl of 10× PCR buffer, 0.375 µl of 50 mM of MgCl_2_, 1 µl of DNA extract and sterile ultra-pure water to a volume of 12.5 µl. All amplifications included positive (slugs of the genus *Arion*) and negative controls. The PCR cycling conditions followed the protocol of Rowson et al. [44]: (i) initial denaturation for 150 s at 94 °C; (ii) 40 cycles of 30 s at 94 °C, 45 s at 47 °C, and 75 s at 72 °C; and (iii) a final extension step at 72 °C for 10 min. Amplification products were visualised under UV light on a 1% Midori Green-stained (Nippon Genetics Europe, Düren, Germany) agarose electrophoresis gel.

Samples that did not yield a visible amplification product were subjected to a new PCR reaction carried out in 11 µl reaction volumes containing 5.5 µl of Qiagen® Multiplex PCR Master Mix containing HotStarTaq® DNA Polymerase, Multiplex PCR Buffer with 6 mM MgCl_2_ and dNTP mix (Qiagen, Venlo, The Netherlands), 1 µl of 2 µM of each primer, 1 µl of DNA extract and sterile ultra-pure water to a volume of 11 µl. All amplifications included positive (slugs of the genus *Arion*) and negative controls. The PCR cycling conditions were: (i) initial denaturation for 900 s at 95 °C; (ii) 40 cycles of 30 s at 94 °C, 30 s at 45 °C, and 60 s at 72 °C; and (iii) a final extension step at 72 °C for 10 min. Amplification products were visualised under UV light on a 1% Midori Green-stained agarose electrophoresis gel.

PCR amplicons were purified using the ExoSAP^TM^ mixture (Thermo Fisher Scientific) according to the manufacturer’s instructions. DNA sequencing reactions (both directions) were performed using BigDye^TM^ Terminator v3.1 Cycle Sequencing Kit (Thermo Fisher Scientific) and an ABI 3130xl capillary DNA sequencer.

DNA sequences were checked, assembled, corrected and trimmed using Geneious Prime (https://www.geneious.com/). Using the Basic Local Alignment Search Tool for nucleotides (BLASTn) of GenBank, the *16S* (*n* = 29) and *cox*1 (*n* = 31) sequences generated were compared to those in GenBank and the Barcode of Life Data-Identification System (IDS-BOLD) in BOLD, respectively. *16S* and *cox*1 sequences were subsequently aligned separately in Geneious Prime. Each dataset included all generated sequences, and a selection of sequences (depending on the number of available reference sequences) of congeneric species retrieved from GenBank and BOLD. Geneious Prime was also used to infer amounts of sequence divergence by calculating p- and Tamura-Nei distances based on pairwise deletion of indels and to reconstruct Neighbor-Joining (NJ) trees. The reliability of tree nodes was assessed by bootstrapping with 1000 replicates. *Boettgerilla pallens* Simroth, 1912 (Boettgerillidae) and *Theba pisana* (Müller, 1774) (Helicidae) were used as outgroups for NJ trees of slugs and snails, respectively. DNA species identifications were based on percentage of sequence homology and the NJ-tree clustering position of the generated sequences relative to GenBank or BOLD reference sequences.

### Detection of metastrongyloid larvae in gastropods

Gastropods were removed from the freezer, left to thaw at room temperature and processed by artificial digestion as previously described with slight modifications [45]. Briefly, each gastropod was weighed (after removal of the shell in the case of snails), minced with a scalpel blade and placed in an Erlenmeyer flask with digestive fluid that contained 0.05 g pepsin 2000FIP, 0.04 g NaCl, 0.13 ml HCl and 5.5 ml tap water per gram of tissue. The samples were digested in a magnetic stirrer at 1000× *rpm* and at 34–37 °C for 75 min. Subsequently, the digestion fluid was poured through a 180 µm sieve in a cone-shaped glass recipient of 1 l to which tap water was added to stop the digestion procedure. The recipient was left for at least 3 h or overnight in the fridge, to allow precipitation. The supernatant was discarded, and the precipitate was transferred to conical glass tubes and centrifuged at 200× *g* for 5 min. The supernatant was discarded, and the precipitate was examined under a light microscope at 45×, 100× and 400× magnifications. Metastrongyloid larvae were identified according to morphological and morphometric characteristics [10, 18, 28, 29, 46]. The larvae were collected individually under a stereomicroscope and stored per gastropod specimen and per species in Eppendorf tubes with absolute ethanol for subsequent DNA-based species identification.

To confirm the identity of the larvae, a triplex semi-nested PCR was performed as previously described [47]. DNA extraction was carried out using a commercial kit (i.e. Qiagen Stool Mini Kit, Qiagen, Hilden, Germany). Briefly, the first step with universal primers NC1 and NC2 was performed. In the second step primers specific for *A. abstrusus* (AabFor: 5'-GTA ACA ACG ATA TTG GTA CTA TG-3'), *T. brevior* (TbrFor: 5'-CGG TGA TTG ATA ATG ATG CAT-3'), and *A. chabaudi* were used in combination with NC2 to achieve specific amplifications of ∼326-bp-, ∼520-bp-, and ∼200-bp-long fragments, respectively. A negative-control sample containing all of the reaction reagents with sterile distilled water to substitute for the template was added to each PCR run. All amplifications have been carried out as previously described [47]. Amplicons were electrophoresed on a 1.8% (wt/vol) agarose gel, stained with GelRed 10,000× (Biotium Inc., Fremont, USA).

The amplicons were purified using a QIAquick gel extraction kit (Qiagen) and then sequenced directly sing BigDye Terminator v.3.1 chemistry (Applied Biosystems, Massachusetts, USA). Sequences were determined in both strands, aligned, and then compared with those of metastrongyloids available in GenBank using BLAST (http://www.ncbi.nlm.nih.gov/BLAST).

## Results

Seven out of 50 (14%) gastropods, i.e. 4 snails and 3 slugs, were found infected with feline metastrongyloids (Table 1). More precisely, 1 out of 8, 1 of 7 and 2 of 7 snails from Kozani, Vyronia and Koronia Lake, and 1 out of 11 and 2 of 5 slugs from sites Kerkini Lake and Volvi Lake, respectively, were found infected.

**Table 1:** Species of infected gastropods the deposited *cox*1 and *16S* GenBank accession numbers, area of collection, species of parasites, larval stage and number of larvae in each gastropod, and area of gastropods’ collection

Gastropod species (GenBank ID: *cox*1/*16S*)	Locality	Coordinates	Altitude (masl)	Parasite species	Life stage
Snails					
*Helix lucorum* (MT293861/MT298682)	Kozani	40.28'N, 21.78'E	710	*Aelurostrongylus abstrusus*	L3 (*n* = 4)
*Massylaea vermiculata* (MT293859/MT298680)	Vyronia	41.26'N, 23.25'E	9	*Aelurostrongylus abstrusus*	L3 (*n* = 10)
*Helix lucorum* (MT293862/–)	Lake Koronia	40.70'N, 23.18'E	100	*Angiostrongylus chabaudi*	L3 (*n* = 5)
*Massylaea vermiculata* (MT293860/MT298681)	Lake Koronia	40.70'N, 23.18'E	100	*Angiostrongylus chabaudi*	L3 (*n* = 58)
Slugs					
*Tandonia* cf. *sowerbyi* (MT293863/MT298683)	Lake Kerkini	41.23'N, 23.09'E	35	*Troglostrongylus brevior*	L2 (*n* = 1); L3 (*n* = 10)
*Limax* cf. *conemenosi* (MT293864/MT298684)	Lake Volvi	40.65'N, 23.49'E	55	*Angiostrongylus chabaudi*	L1 (*n* = 5); L3 (*n* = 4)
*Limax* cf. *conemenosi* (MT293865/MT298685)	Lake Volvi	40.65’N, 23.49'E	55	*Angiostrongylus chabaudi*	L3 (*n* = 2)

*Abbreviations*: masl, meters above sea level; L1, first-stage larvae; L2, second-stage larvae; L3, third-stage larvae

### Identification of gastropods and infection detected in each gastropod

One snail from Vyronia and one from the area of Lake Koronia were identified as *Massylaea vermiculata* (Müller, 1774). One snail from Kozani and one collected near Lake Koronia were identified as *Helix lucorum* Linnaeus, 1758. One slug collected near Lake Kerkini was identified as *Tandonia* cf. *sowerbyi* (Férussac, 1823) by its external morphology, but its DNA identification was feasible only at the genus level (*Tandonia* sp.), because the sequence clustered significantly within the *Tandonia* cluster in the NJ tree (not shown). Yet, there was no obvious match with any specific *Tandonia* reference sequence, since the closest best-match sequence similarities were only 88–94% (three *Tandonia* species). Similarly, 2 slugs collected near Lake Volvi were identified as *Limax* cf. *conemenosi* Boettger, 1882 by their external morphology, but their DNA-based identification was feasible only at the genus level (*Limax* sp.), because of significant clustering within the *Limax* cluster in the NJ tree (not shown), but without a clear match with any specific *Limax* reference sequence, except for a 100% *cox*1 sequence match with “*Limax* sp. Balkan”. The available specimens were juveniles, thus morphological examination of the reproductive system that would allow identification at the species level was not possible. DNA-based species identifications were consistent between *cox*1 and *16S* in terms of best matching with reference sequences in GenBank and/or BOLD, supported branching NJ clustering NJ-trees and the morphological corroboration. The obtained *cox*1 and *16S* sequences of the identified gastropods have been deposited in the GenBank database under the accession numbers shown in Table 1.

Larvae of *A. abstrusus* were found in *M. vermiculata* (*n* = 10, L3) from Vyronia and *H. lucorum* (*n* = 4, L3) collected from Kozani. *Troglostrongylus brevior* (*n* = 1 L2 and *n* = 10 L3) were found in *T.* cf. *sowerbyi* collected from Kerkini Lake. *Angiostrongylus chabaudi* were found in *L.* cf. *conemenosi* (*n* = 5 L1 and *n* = 4 L3) collected from the area of Lake Volvi and in *H. lucorum* (*n* = 5 L3) and *M. vermiculata* (*n* = 58 L3), from Lake Koronia (Table 1).

### Identification of metastrongyloid nematodes

**Fig. 2:**
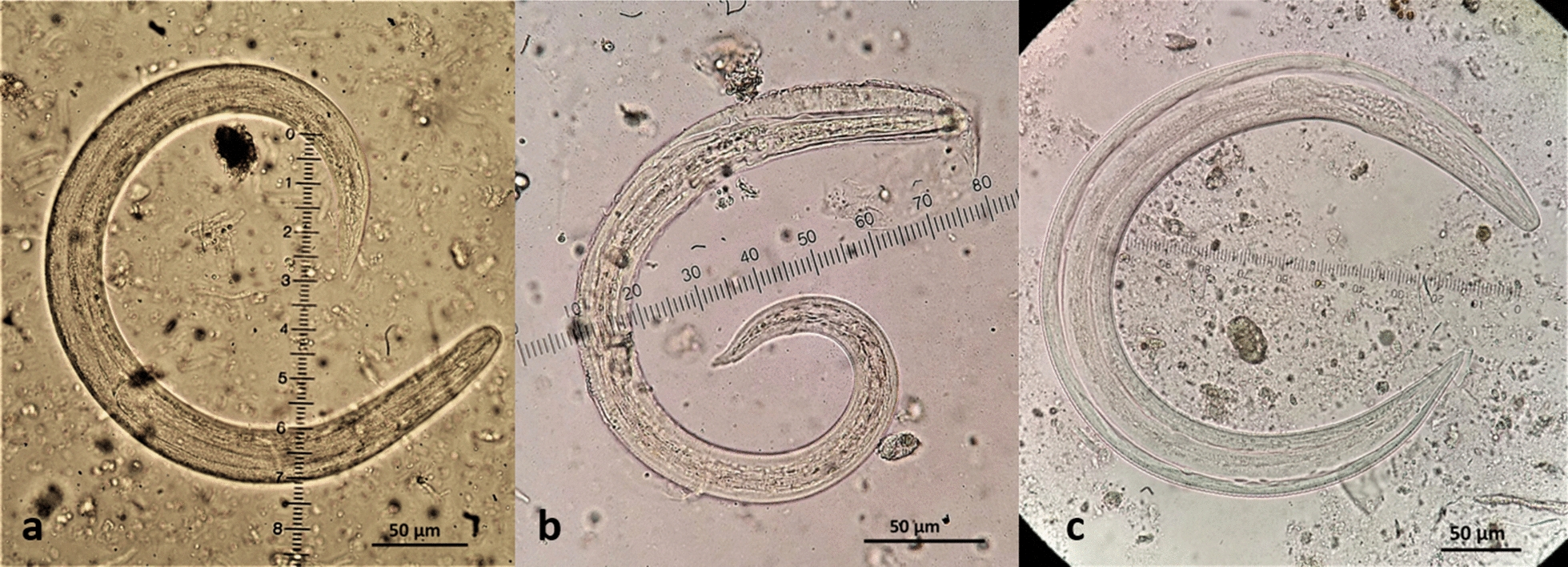
Third-stage larvae (L3) of feline cardiopulmonary nematodes found in naturally infected intermediate gastropod hosts in Greece. **a***Aelurostrongylus abstrusus*. **b***Troglostrongylus brevior*. **c***Angiostrongylus chabaudi*

**Fig. 3:**
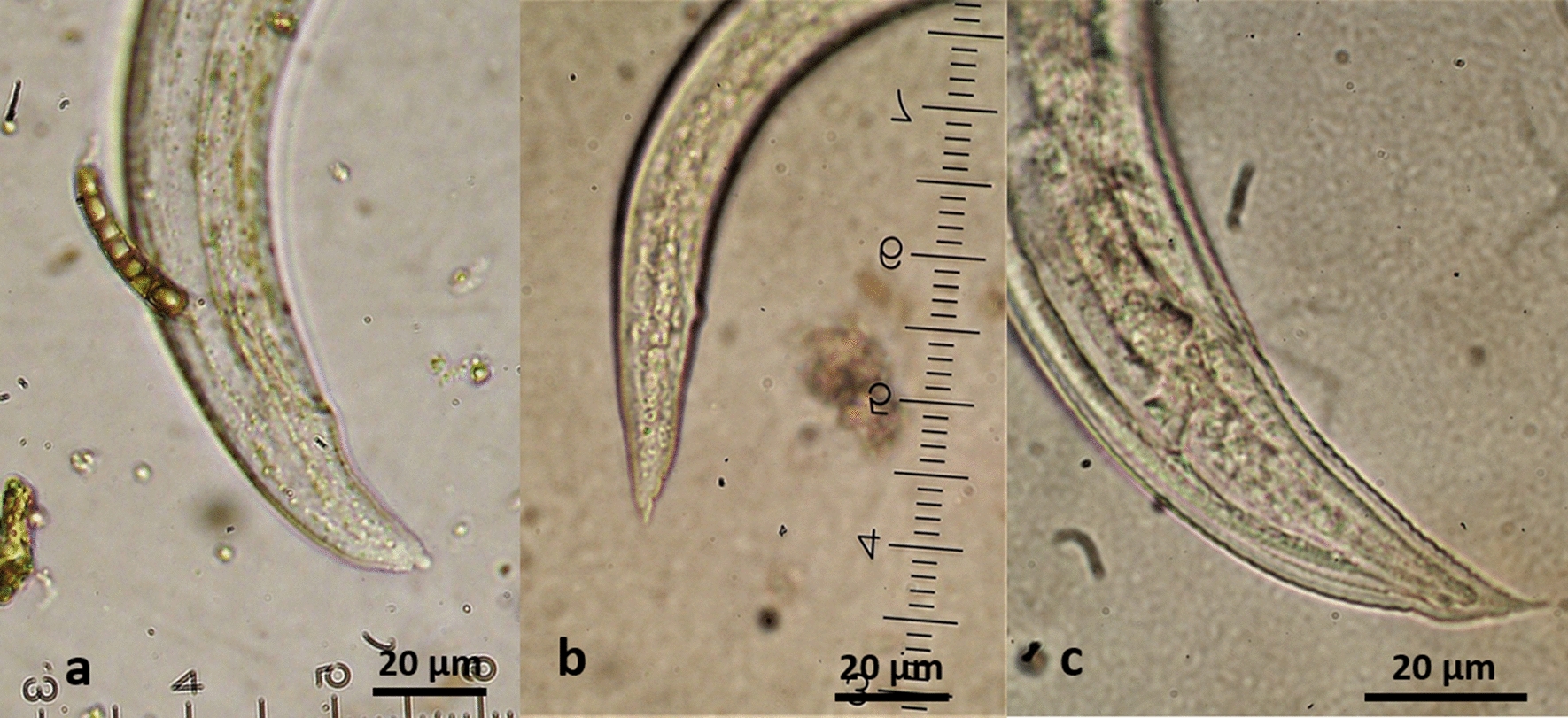
Morphology of the posterior region of the third-stage larvae (L3) of feline cardiopulmonary nematodes found in naturally infected intermediate gastropod hosts in Greece. **a***Aelurostrongylus abstrusus* with a terminal knob. **b***Troglostrongylus brevior* with one caudal and two ventral notches and a small slightly rounded tip of tail. **c***Angiostrongylus chabaudi* with two caudal notches and an acute tip of tail

*Aelurostrongylus abstrusus* L3 (Fig. 2) were C-shaped, 444–608 μm in length with maximum body width of 25–33 μm. The length of the oesophagus was 175–195 μm with the distance of the nerve-ring from the anterior extremity measuring 75–85 μm. The posterior end was conical with a rounded projection, the characteristic knob of *A. abstrusus* L3 (Fig. 3). The distance from the anus to the tip of the knob was 28–35 μm. The genital primordium was observed at 162–193 μm from the caudal extremity and the excretory pore was approximately at 91–94 μm from the cephalic extremity of the larvae (Table 2).

**Table 2:** Measurements (in μm) of the larval stages of feline cardiopulmonary nematodes in gastropods in Greece, presented with corresponding ranges from previous reports

Characteristics	*Aelurostrongylus abstrusus*			*Troglostrongylus brevior*						*Angiostrongylus chabaudi*	
	E^a^	N^b^	P (*n* = 14)	E^c^		N^d^		P (L2 = 1, L3 = 10)		E^e^	P (*n* = 69)
	L3	L3	L3	L2	L3	L2	L3	L2	L3	L3	L3
Total body length	459–670	330–635	444–608	330–399	417–450	nr	nr	330	428–497	580–710	444–868
Maximum body width	25–38	22–38	25–33	22–28	19–23	nr	nr	20	20–25	28–43	32–40
Oesophagus length	194–221	130–215	175–195	110–125	121–145	nr	nr	160	125–158	213–277	175–250
Nerve-ring to anterior extremity	75–80	40–72	75–85	nv	60–67	nr	nr	nv	75–80	76–102	88–110
Anus to posterior extremity	34–44	20–35	28–35	35	30–42	nr	nr	35	40–43	27–47	43–55
Primordium to posterior extremity	126–265	150–180	162–193	nv	168	nr	nr	nv	167–178	181–222	90–168
Excretory pore to anterior extremity	87–102	90–99	91–94	nv	50–79	nr	nr	nv	85–92	84–119	93–112

^a^References [10, 28, 29, 32, 34, 46–48]

^b^References [31, 35, 36]

^c^References [10, 28]

^d^References [38, 39]

^e^Reference [44]

*Abbreviations*: E, experimental infection; N, natural infection; P, present study (number of larvae measured); nv, not visible; nr, not reported; L2, second stage larvae; L3, third-stage larvae

**Fig. 4:**
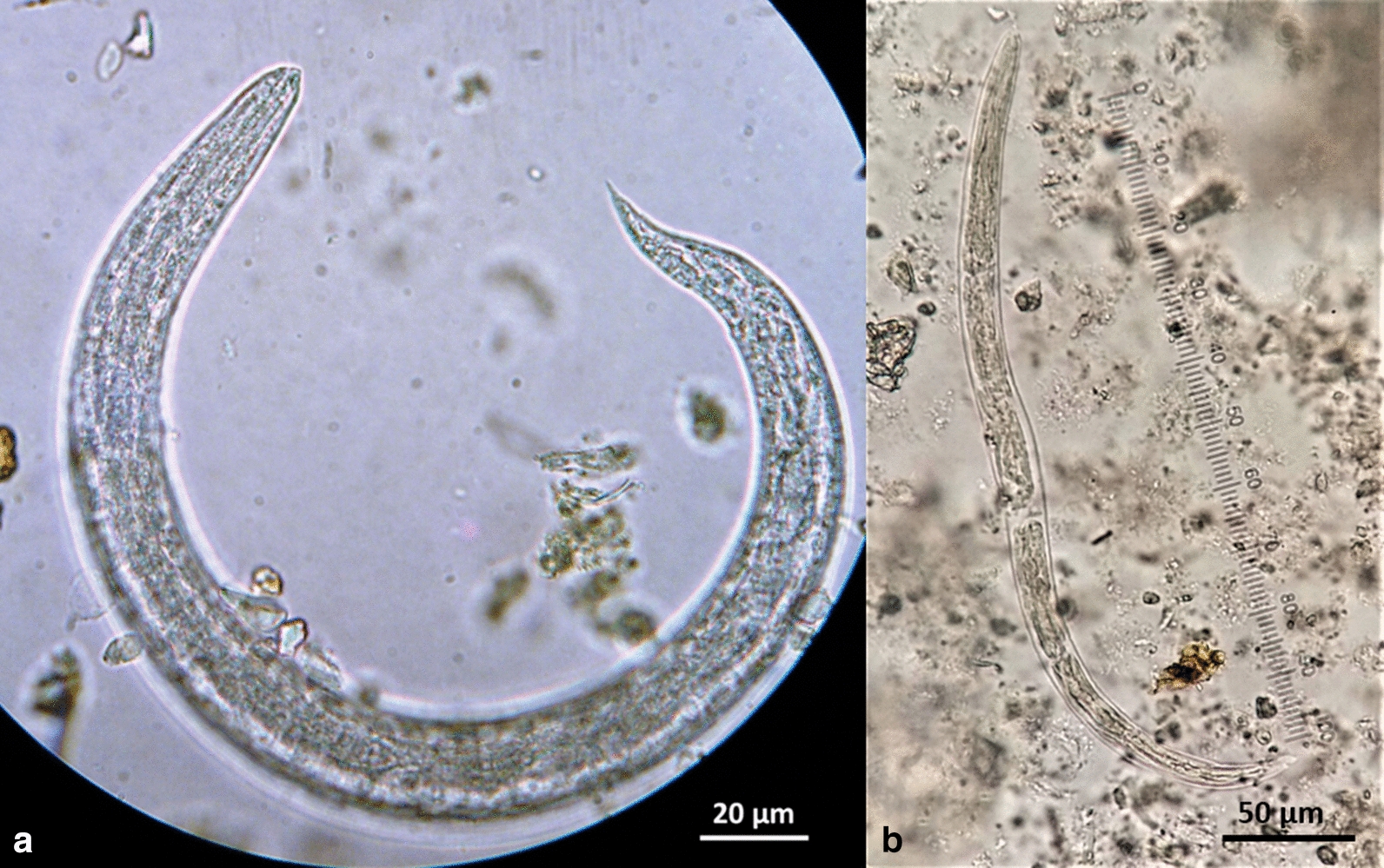
*Troglostrongylus brevior* (L2) (**a**) and *Angiostrongylus chabaudi* (L1) (**b**) found in naturally infected gastropods in Greece

The second-stage larva (L2) of *T. brevior* (Fig. 4) found in *T.* cf. *sowerbyi* was C-shaped, covered with the cuticle of L1, measuring 330 μm in length and 20 μm in width. The oesophagus was 160 μm and tail (anus to posterior extremity) was 35 μm in length. The anterior extremity was narrow, widening progressively towards the middle of the body that showed granular content. Third-stage larvae (L3) (Fig. 2) were also C-shaped, measured 428–497 μm in length, with maximum body width of 20–25 μm, observed at about mid-body. The oesophagus was 125–158 μm long, the nerve-ring was located at 75–80 μm from the anterior extremity and the anus to tail distance was 40–43 μm. Genital primordium was situated at 167–178 μm from the posterior extremity while the excretory pore was located slightly posterior to the nerve-ring, at 85–92 μm from the anterior extremity (Table 2). The caudal region was slightly bent dorsally and ended in three terminal notches (one caudal and two ventral) and a small, slightly rounded appendage (Fig. 3).

Five *A. chabaudi* larvae found in *L.* cf. *conemenosi* were in the first stage (L1, Fig. 3), measuring 310–345 μm in length and 15–19 μm in width. The anus was placed at 33–38 μm from the posterior extremity that showed the typical L1 kinked tail morphology with a subterminal dorsal spine separated by a moderately wide and distinct notch. The oesophagus measured 100–160 μm in length and the nerve-ring was evident at 43–80 μm from the anterior extremity. The excretory pore was situated slightly posterior to the nerve-ring at 75–88 μm from the anterior extremity. The genital primordium was small and oval in shape situated at 100–113 μm from the posterior extremity. The L3 of *A. chabaudi* (Fig. 2) were 444–868 μm in length and 32–40 μm in width, with a transversally striated cuticle. The oesophagus was 175–250 μm in length and the nerve-ring was located at 88–110 μm from the anterior extremity. The excretory pore was situated slightly posterior to the nerve-ring, at 93–112 μm from the anterior extremity. The genital primordium was located at 90–168 μm from the posterior extremity of the parasite (Table 2). The anus to tip of the body distance was 43–55 μm. The posterior end showed a quite sharp final tip, and two caudal notches (Fig. 3).

Table 2 provides the measurements of all developmental stages of the nematode species reported here in comparison with previously reported data [10, 28, 29, 31, 32, 34–36, 38, 39, 46, 48–50].

The identity of the larvae microscopically identified as *A. abstrusus*, *T. brevior* and *A. chabaudi* was molecularly confirmed by the species-specific PCRs and sequencing. Specifically, the ITS2 regions of *A. abstrusus*, *T. brevior* and *A. chabaudi* here generated displayed 100% homology with sequences available on GenBank (GenBank: DQ372965.2, KM506759.1 and KM216825.1, respectively).

## Discussion

The results of the present study have been preliminarily presented at the International Congress on the Zoogeography and Ecology of Greece and Adjacent Regions in June 2019 [51]. Subsequently, the presence of *T. brevior* in terrestrial gastropods in Europe was confirmed in Austria, though from a different intermediate slug host [39]. The findings on *A. chabaudi*, including the description of its larval stages are relevant, because the life-cycle of *A. chabaudi* is still poorly known, and the present study confirms for the first time that gastropods are competent intermediate hosts in natural conditions.

Gastropods act as intermediate hosts for various parasites [27, 52], including feline cardiopulmonary nematodes. These nematodes have been successfully developed in snails or slugs in a series of experimental infections (Table 2). However, experimental infection and observation of parasite development in tissues of gastropods do not necessarily indicate that the specific gastropod species can act as a natural intermediate host or have any epizootiological importance. Thus, the current knowledge on gastropod species involved in the transmission of feline metastrongyloid parasites in natural conditions is limited. Nevertheless, natural infections with *A. abstrusus* have been occasionally reported (Table 2). In the case of *T. brevior* there are recent reports of natural infection in *Lissachatina fulica* and *Arion vulgaris* in Colombia and Austria, respectively [38, 39], although no morphological description of the isolated parasites is provided. Hence, the present results add new gastropod species to the natural intermediate host spectrum of the three nematodes.

As expected, the circulation of gastropod transmitted nematodes depends on the (micro-) distributional dynamics of their intermediate hosts [29, 53]. In some cases, this may explain the patchy distribution of snail-borne nematodes, such as *Angiostrongylus vasorum* [54]. In this context, it is important to assess and understand some basic biological characteristics of the gastropods in question. *Helix lucorum* and *M. vermiculata*, which were here found to harbour both *A. abstrusus* and *A. chabaudi*, are widespread over southern Europe and have been introduced in several regions within and beyond their native area, where they may be found in natural and anthropized habitats [55–57]. As these snails are synanthropic species, are being cultured (heliciculture), consumed and traded [58], they may be easily and repetitively transferred, potentially contributing to the spreading and establishment of metastrongyloids in new areas. Subsequently, all regions where *M. vermiculata* (Europe, Asia, Africa, USA and Australia) and *H. lucorum* (southern Europe and the south and west of the Caspian Sea) have been recorded may be at risk of *A. abstrusus* and *A. chabaudi* infections in felids. While to date *A. abstrusus* has been reported from many parts of the world, i.e. Europe, Kenya [59], Australia [60], South America [32, 61, 62], Israel [63] and the USA [64], *A. chabaudi* has only been reported from Bulgaria, Bosnia and Herzegovina, Germany, Greece, Italy, Romania and Spain [2, 5, 6, 18–20, 65]. However, the dispersal capacity of the intermediate hosts reported here (two of which are commonly introduced elsewhere in Europe), makes it plausible that *A. chabaudi* may expand beyond its current distribution in Europe.

Both *H. lucorum* and *M. vermiculata* have a life span of at least two to five years, but *H. lucorum* may become much older (up to > 14 years) [66–69]. Thus, these snails may accumulate and maintain the nematode infective L3 stage for long periods, as for example, *A. abstrusus* L3 can survive for up to two years in infected gastropods [70]. According to the authors’ experience *A. chabaudi* L3 remain alive in gastropods preserved at − 20 °C for at least two years (unpublished data), thus low temperatures during the hibernation period of the snails most probably do not affect the vitality of the larvae. Thus, snails may maintain and conserve the infective stage of the parasites from one activity season, throughout hibernation, to the next one. Interestingly, it has been recently shown under experimental conditions that natural hibernation of snails may enhance the parasitic burden, at least for *T. brevior* infective larvae [71]. It would be interesting to document for how long L3 can remain alive and infective inside the gastropods in natural conditions, as this would provide information about the potential of the parasites to accumulate in the gastropod population of a given environment. Depending on the gastropod species and the environmental temperature, it takes around two to three weeks for *A. abstrusus* L1 to develop to L3 [10, 28, 29] and 10–30 days for *A. chabaudi* L1 to develop to L3 [46]. Given this variation in developmental times and considering that the development of larvae depends on the age and size of the gastropods [28, 29, 36, 49], it is not surprising that some larval measurements reported here differ significantly from those published elsewhere (Table 2).

The slugs *T.* cf. *sowerbyi* and *L.* cf. *conemenosi*, which were infected with *T. brevior* and *A. chabaudi*, respectively, are neither consumed by man nor cultured, but have a synanthropic biology. As such they are easily transported by man (e.g. *via* objects, media of transportation, goods) and introduced in areas beyond their native ranges [72, 73]. *Tandonia sowerbyi* is a eurytopic species that lives in a wide variety of habitats where it hides under shrubs, stones, wood and soil litter. It prefers areas free of tree cover, but it can also be abundant in wastelands, gardens and farm-lands [72–74], i.e. environments that meet the characteristics of the wildcat-domestic cat sympatric habitats. It is native and very common in Greece and has been introduced in various areas of the world [72, 75]. Although more gastropod species likely act as intermediate host of *T. brevior*, the distribution of *T. sowerbyi* in southern-eastern Europe, and South America overlaps that of felid *T. brevior* infections hitherto reported [13, 15, 22]. Apart from the suitable gastropod species, the development of *T. brevior* L1 to L3 depends on the environmental temperature, varying accordingly from eight to 40 days, thus even the smallest temperature change could alter the distribution of gastropod borne diseases [76–79]. Consequently, global warming may facilitate the expansion of cardiopulmonary nematodes in more northern geographical areas, both by permitting the establishment of suitable gastropod species in these areas where they are currently absent [36, 76] and by shortening the period of extrinsic parasite development. The range of *L. conemenosi* that is native to Greece extends to Bulgaria, Republic of North Macedonia and Albania [73, 74, 80, 81]. This species lives mainly in rural areas [73, 74, 80], yet in the present study it was found in a wild habitat close to Lake Volvi. The occurrence of this species in natural and anthropized environments may facilitate the spread of metastrongyloid nematodes from wildcats to domestic cats and *vice versa*.

Among the localities where wildcats and domestic cats occur in sympatry in the present study, the wetland areas of lakes Kerkini, Koronia and Volvi (Fig. 1) are listed in Natura 2000 [82] a geographical network of protected areas where rare and threatened wildlife species can breed and rest. The areas around these lakes are typical wildcat habitats with a well-established population of wildcats [83, 84]. In the same areas there are villages, where domestic cats either free roam or have outdoor access. Similarly, in more urbanised environments such as the areas near the city of Kozani and the village of Vyronia (Fig. 1), wildcats searching for food may approach areas close to human activity, where free roaming domestic cats are present. Under the conditions in all five areas of the present study, wildcats and domestic cats live in sympatry [85, 86], thus creating the conditions for the spread of parasites harboured by one group of animals to the other [6, 21, 87]. A spillover of this kind has been demonstrated for *A. vasorum*, as in areas where foxes (the natural host reservoir) are heavily parasitized, wolves and dogs are also infected [9, 88–93]. As infections with *A. abstrusus*, *T. brevior* and *A. chabaudi* were recently reported in *F. silvestris* in areas close to human activity [21, 94], the epizootiological pressure to domestic cats is a realistic scenario.

Reports of domestic cats co-infected with *A. abstrusus*, *T. brevior* and *A. chabaudi* [6, 12, 13] support the existence of a shared transmission pattern for these nematodes among their definitive (domestic cats and wildcats) and intermediate hosts. In wildcat habitats these parasites circulate in the sylvatic life-cycle among wildcats, intermediate and paratenic hosts [87] (AD, unpublished data), rendering these areas a possible source of infection for domestic cats that live in proximity.

The present data were obtained from a limited number of specimens and do not permit solid conclusions about the prevalence of infection of gastropods by feline cardiopulmonary nematodes and the corresponding pressure of parasite spillover in areas of wildcat and domestic cat sympatry. Yet, for *A. chabaudi*, it has been recently suggested that domestic cats are, for the time being, not in particular risk [95]. However, the data presented herein are of importance under biological and epizootiological standpoints. Information about the suitability of various intermediate hosts and knowledge about their distribution, biological features and abundance is important in order to predict the expansion of cardiopulmonary parasites in felids and the possible risk of parasite spillover. Finally, the prevalence of infection of gastropods that act as intermediate hosts of feline metastrongyloids in the areas of study remains to be assessed and would provide additional important information on the epizootiology of these parasites.

## Conclusions

To the best of our knowledge, the present study offers for the first time conclusive evidence that gastropods act as intermediate hosts for *A. chabaudi* under natural conditions. Furthermore, developmental stages of *T. brevior* are reported and morphologically described for the first time in gastropods in Europe and infected intermediate gastropod hosts of *A. abstrusus* were for the first time detected in Greece. The biological characteristics and distribution patterns of these gastropods suggest that these three parasites may further expand to areas where infected felids have not yet been reported. Finally, this study suggests that these parasitic nematodes may cross-infect wildcats and domestic cats in areas were these animals live in sympatry.

## Data Availability

All data generated or analysed during this study are included in this published article. Representative sequences were deposited in the GenBank database under the accession numbers MT293861, MT298682, MT293862 (*Helix lucorum*), MT293859, MT298680, MT293860, MT298681 (*Massylaea vermiculata*), MT293863, MT298683 (*Tandonia* cf. *sowerbyi*), MT293864, MT298684, MT293865, MT298685 (*Limax* cf. *conemenosi*). Any additional data (photographs etc.) are available upon request.

## Abbreviations

BOLD: barcode of life data
BLASTn: nucleotide basic local alignment search tool
*cox*1: cytochrome *c* oxidase subunit 1
dNTP: deoxynucleoside triphosphates
IDS: identification system
L1: first-stage larva
L2: second-stage larva
L3: third-stage larva
mtDNA: mitochondrial DNA
NJ: neighbor-joining
UV: ultraviolet
